# Evaluation of sun holiday, diet habits, origin and other factors as determinants of vitamin D status in Swedish primary health care patients: a cross-sectional study with regression analysis of ethnic Swedish and immigrant women

**DOI:** 10.1186/1471-2296-14-129

**Published:** 2013-09-03

**Authors:** Anne Björk, Åsa Andersson, Gunnar Johansson, Karin Björkegren, Annika Bardel, Per Kristiansson

**Affiliations:** 1 Department of Public Health and Caring Sciences, Family Medicine and Preventive Medicine unit, Uppsala University, Box 564, Uppsala SE-751 22, Sweden; 2 Gottsunda Primary Health Care Centre, Box 25024, Uppsala SE-750 25, Sweden

**Keywords:** Vitamin D, Sun habits, Immigrant, Women, Primary health care

## Abstract

**Background:**

Determinants of vitamin D status measured as 25-OH-vitamin D in blood are exposure to sunlight and intake of vitamin D through food and supplements. It is unclear how large the contributions are from these determinants in Swedish primary care patients, considering the low radiation of UVB in Sweden and the fortification of some foods. Asian and African immigrants in Norway and Denmark have been found to have very low levels, but it is not clear whether the same applies to Swedish patients. The purpose of our study was to identify contributors to vitamin D status in Swedish women attending a primary health care centre at latitude 60°N in Sweden.

**Methods:**

In this cross-sectional, observational study, 61 female patients were consecutively recruited between January and March 2009, irrespective of reason for attending the clinic. The women were interviewed about their sun habits, smoking, education and food intake at a personal appointment and blood samples were drawn for measurements of vitamin D and calcium concentrations.

**Results:**

Plasma concentration of 25-OH-vitamin D below 25 nmol/L was found in 61% (19/31) of immigrant and 7% (2/30) of native women. Multivariate analysis showed that reported sun holiday of one week during the last year at latitude below 40°N with the purpose of sun-bathing and native origin, were significantly, independently and positively associated with 25-OH-vitamin D concentrations in plasma with the strongest association for sun holiday during the past year.

**Conclusions:**

Vitamin D deficiency was common among the women in the present study, with sun holiday and origin as main determinants of 25-OH-vitamin D concentrations in plasma. Given a negative effect on health this would imply needs for vitamin D treatment particularly in women with immigrant background who have moved from lower to higher latitudes.

## Background

Vitamin D is known to play a major role in maintaining bone health and calcium homeostasis. More recently, research has revealed the possibility of additional important roles of the vitamin, in connection with a variety of health problems such as cancer and infectious, cardiovascular and autoimmune diseases [1].

The supply of vitamin D to the body is through food intake, supplements and from the endogenously production through ultraviolet radiation B of the skin. Natural food sources of vitamin D are fatty fish, cod liver oil, and to a lesser extent egg yolk, some mushrooms, and meat [2,3].

In countries where ultraviolet radiation B is low, the population is likely to be dependent on dietary sources of vitamin D to meet their biological needs during part of the year. Therefore, the authorities of some countries at high latitudes require fortification of certain food products.

Plasma concentrations of 25-OH-vitamin D (25(OH)D), with a half-life of 1-2 months, are believed to adequately reflect the vitamin D status as regards production, absorption, and storage [4].

The relative importance of these different sources and possible interaction mechanisms for vitamin D status in primary health care at more northerly higher latitudes has not to our knowledge, been previously investigated.

The aim of this study was to investigate plasma concentrations of 25(OH)D and to evaluate possible factors influencing the concentrations among women seeking primary health care for various reasons.

## Methods

The study was a cross-sectional observational study performed during 18 days between January and March 2009 at a primary health care centre in a district of the city of Uppsala, Sweden, where half of the residents have an immigrant background, with two thirds originating from the Middle East [5].

Patients living in Sweden, attending the primary health care centre, with appointments to visit a physician or a dietician, were invited to participate, irrespective of the reason for the appointment. Following the recruitment appointment a study appointment was scheduled within the recruitment period. Inclusion criteria were female sex, age 18 to 75 years, origin from Africa, Asia or Sweden. Exclusion criteria were current pregnancy, current breastfeeding or current treatment with vitamin D. In total, 38 immigrant patients (registered as immigrants) and 30 Swedish patients (registered as native women) were recruited. Four of the immigrants declined participation, owing to lack of time or interest and further three did not turn up for the study appointment. All native women who were invited to participate agreed and all returned for the study appointment. In total, 31 immigrants and 30 native women were thus included. Those who were interested in participating received both oral and written information and signed a written consent form.

The most common reasons for attending the primary health care centre were internal medicine issues. There was no difference regarding the medical reasons for the appointment between the two groups.

At the study appointment, a general practitioner and a dietician met each patient together. Women not fluent in Swedish received help filling out the questionnaire. In six cases a professional interpreter was used and in one case, the husband was present and helped to translate. Information on their general health, present smoking habits, education, eating habits, holidays in the sun, use of sunscreen and skin reactivity was obtained by structured written questionnaires and interview. Wearing a veil was noted.

Since vitamin D production in the skin is affected by the degree of pigmentation of the skin, photosensitivity has been estimated by Fitzpatrick sun-reactive skin-type classification which includes six different skin types [6]. Skin types I to III include light skin, while skin types IV to VI include darker skin. Therefore, participants completed a detailed questionnaire covering sun-related behaviour. The results were assessed by the physician (A.B.). Sun holiday was defined as a journey of at least one week during the past year, to a southern (below 40°N) latitude with the aim of sunbathing. Journeys with the aim of staying mainly indoors, e.g. visiting relatives, were not counted.

Intake of vitamin D from food and supplements was estimated using a new semi-quantitative food frequency questionnaire (FFQ), designed for this study. This FFQ consisted of 15 foods and 8 frequencies and aimed to assess the intake during the previous two to three months. The FFQ included foods containing vitamin D naturally, and foods fortified with vitamin D (low fat milk, milk products and margarine). The feasibility of the FFQ was checked with the patients and all items in the FFQ were discussed together with each participant during the appointment.

Height and weight were measured and Body Mass Index (BMI, kg/m^2^) was calculated. Blood samples were drawn between 09.00 and 16.00 without previous fasting, by venepuncture into an EDTA tube and centrifuged immediately. Plasma samples were frozen at -25°C and sent to the laboratory. Concentrations of 25(OH)D were analysed using chemiluminiscent immunoassay [7]; the lowest possible value detectable was 10 nmol/L. Concentrations of protein-bound calcium in plasma were also analysed. All analyses were performed at the laboratory of Clinical Chemistry, Uppsala University Hospital, Uppsala, Sweden.

The study was approved by the local ethics committee at Uppsala University (Local ID number 2008:359).

### Statistical analyses

Non-parametric tests were used since the data was not normally distributed. Correlation was analysed with Spearman’s correlation coefficient and differences between the groups in continuous variables with Wilcoxon’s test. Differences in proportions were calculated with the chi-square test. For simple and multiple regression analyses the SAS procedure General Linear Model, a linear regression method not requiring normally distributed data and providing regression as well as unbalanced anova analyses, was used for univariate and multivariate analyses. To avoid model overload stepwise regression as well as backward elimination of non-significant variables were used. In univariate and multiple linear regression analyses the general linear model was used. Both methods yielded the same result and therefore only results based on backward elimination are presented. Continuous variables were age (years), BMI (kg/m^2^), weight (kg), vitamin D intake (food and supplements) (μg/24 hours) and plasma calcium (mmol/L). The categorical variables included origin (immigrant/native), education (<12 years/>12 years), use of sunscreen (no/yes), wearing veil (not observed/observed), sun holiday past year (no/yes), skin type (I-VI) and current smoking (no/yes).

**Figure 1:**
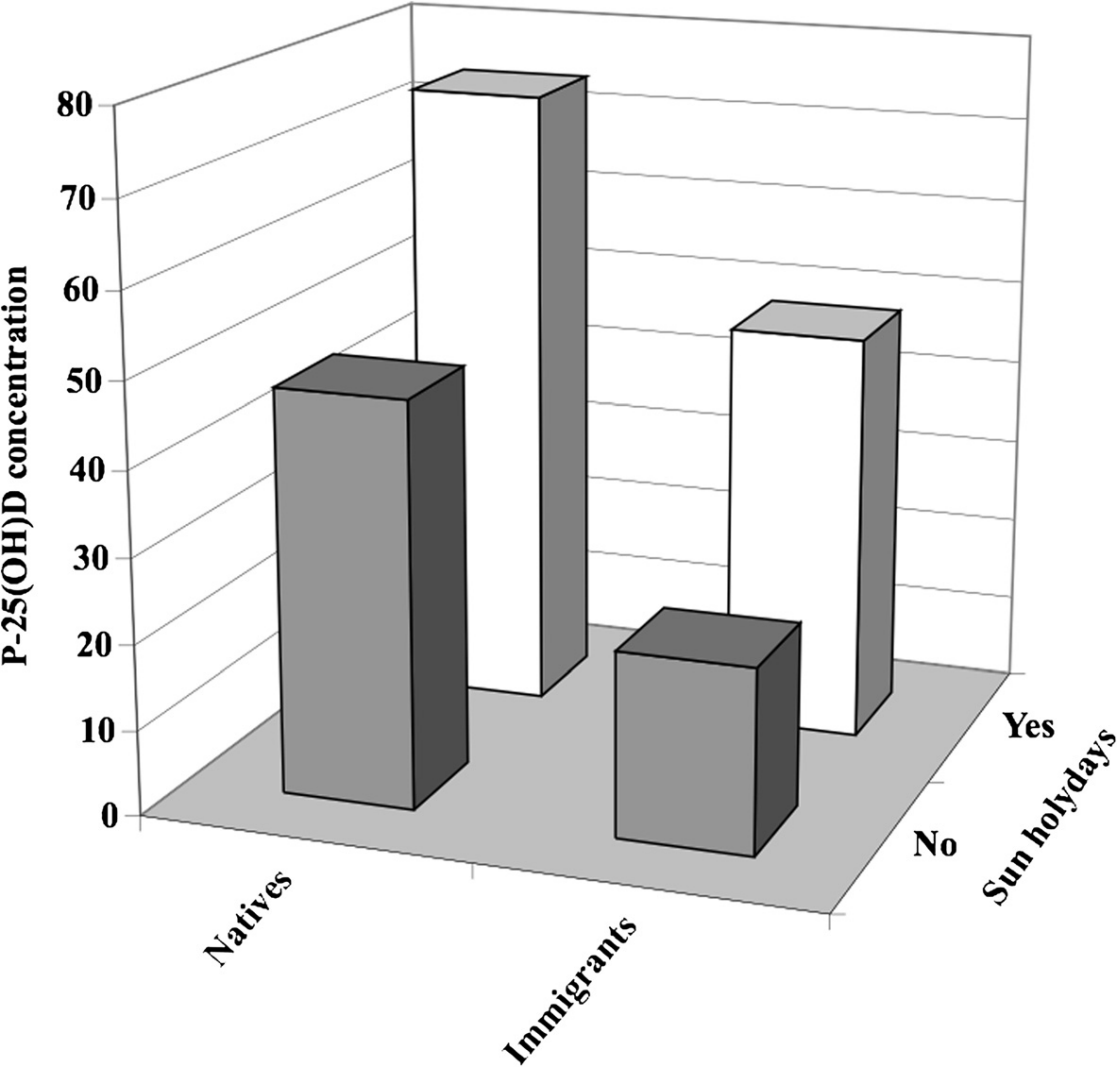
**Plasma-25-OH-vitamin D concentrations (P-25(OH)D) by origin and reported sun holiday.** The general linear regression model was used to compute expected mean plasma concentrations of 25-OH-vitamin D estimates based on origin and reported sun holiday.

For the analyses shown in the regression surface in Figure 1, the general linear regression model was used to compute expected mean plasma concentrations of 25(OH)D estimates based on origin and reported sun holiday. No collinearity problems were stated with variance of inflation <2 in all used factors. P-values less than 5% were regarded as statistically significant. No account was taken of multiple comparisons. Statistical analysis was performed using the SAS program package version 9.2 (SAS Institute, Cary, NC, USA).

An estimation of statistical power was made before the study started to determine the number of patients needed in order to find a statistically significant difference between 25(OH)D in immigrants and Swedes, assuming that 60% of immigrants and 20% of ethnic Swedish women had 25(OH)D < 25 nmol/L. The concentration 25 nmol/was used as a cut-off level since at this level, serum PTH concentrations are raised and high bone turnover has been observed. The power analysis indicated that a sample of 60 women would be sufficient to reveal a statistically significant difference with 90% power using a 2-sided test of significance and p < 0.05.

## Results

Characteristics of participants in the study group are presented in Table 1. The immigrants originated from (were born in): Iraq 8 (7 of them from Kurdistan), Turkey 4, Palestine 4, Somalia 4, Iran 3, Syria 3, Eritrea 2, Jordan 1, Bangladesh 1, and Armenia 1. The immigrant women had lived in Sweden for between 2 and 26 years, with a median of 12 years. They wore veils to a higher extent, had less education, fewer had skin types I-III, reported lower intake of vitamin D in food and supplements, and displayed lower 25(OH)D as compared with the ethnic Swedish women. There were no statistically significant differences in age, weight, BMI, current smoking, sun holiday during the past year, or use of sunscreen between the groups. All women had normal plasma calcium concentrations.

**Table 1:** Characteristics presented in all women and separately by women of ethnic Swedish and immigrant origin

**Characteristic**	**All women (n = 61)**	**Native women (n = 30)**	**Immigrant women (n = 31)**	p*)
Age (years)^1^	39 (29-51)	42 (31-56)	35 (29-49)	0.19
BMI (kg/m^2^)^1^	27 (22-32)	26 (22-32)	28 (24-32)	0.36
Current smoking (%)^2^	12 (20)	6 (20)	6 (19)	1.00
Sun holiday past year (%)^2^	13 (21)	8 (23)	6 (19)	0.76
Time since sun holiday (wks.)^1^	30 (26-38)	31 (17-32)	29 (28-38)	0.68
Observed wearing of veil (%)^2^	11 (18)	1 (3)	10 (32)	0.006
Use of sunscreen (%)^2^	41 (67)	19 (63)	22 (71)	0.52
Education >12 years (%)^2^	37 (61)	22 (73)	15 (48)	0.001
Skin types I-III (%)^2^	44 (72)	28 (93)	16 (52)	0.012
P-calcium (mmol/L)^1^	2.3 (2.3-2.4)	2.3 (2.3-2.4)	2.3 (2.3-2.4)	0.55
Vitamin D intake, food (μg/day)^1^	4.0 (2.5-5.7)	5.1 (4.0-6.4)	3.1 (2.0-4.4)	0.0008
Vitamin D intake, food + supplements (μg/day)^1^	4.0 (2.5-6.1)	5.6 (4.0-8.1)	3.1 (2.0-4.7)	0.0009
25(OH)D (nmol/L)^1^	34.0 (21.8-52.0)	51.5 (39.9-65.0)	22.2 (13.7-28.6)	<0.0001

Median (25th to 75th percentiles) and numbers (%) are presented.

*) p-values refers to the difference between native and immigrant women.

^1^ = p-value calculated with Wilcoxon’s non-parametric test.

^2^ = p-value calculated with chi-square test.

P-calcium = plasma concentration of calcium.

25(OH)D = plasma concentration of 25-OH-vitamin D.

The impact of possible factors influencing 25(OH)D concentrations is displayed in Table 2. In the univariate regression analysis BMI, education, and reported sun holiday were positively associated with 25(OH)D concentrations. Being of immigrant origin and observed wearing of veil were negatively associated with 25(OH)D concentrations.

**Table 2:** Effects of different characteristics on plasma concentration of 25-OH-vitamin D in several simple linear regression analyses and one multiple linear regression analysis (n = 61)

	**Univariate linear regression**			**Multiple linear regression**	
**Variable**	**β-coefficient**	**R**^**2**^	**P**	**β-coefficient**	**p**
Age (years)	0.33	0.03	0.16	-0.03	0.87
BMI (kg/m^2^)	-1.19	0.13	0.005	-0.63	0.08
Native origin	27.8	0.32	<0.001	-22.5	0.0001
Vitamin D intake, f + s (μg/day)	1.6	0.12	0.06	0.14	0.76
Plasma calcium (mmol/L)	41.4	0.02	0.26	28.1	0.30
Education >12 years	8.1	0.08	0.03	-0.02	0.99
Use of sunscreen	-5.7	0.03	0.15	0.98	0.75
Observed wearing of veil	-24.4	0.14	0.03	-7.0	0.33
Sun holiday past year	31.2	0.27	<0.0001	25.2	<0.0001
Skin type	-3.5	0.03	0.15	-1.18	0.58
Current smoking	-9.8	0.02	0.22	-6.6	0.28

R^2^ = 0.62.

f + s = food supplements.

To find factors that independently have an impact on 25(OH)D, a multiple linear regression analysis was performed. Reported sun holiday and ethnic Swedish origin, were positively, significantly and independently associated with 25(OH)D with the strongest association displayed for sun holiday (Table 2).

To illustrate the effect of reported sun holiday and ethnic Swedish origin on 25(OH)D plasma concentrations, the linear regression model was used to compute expected mean 25(OH)D (Figure 1). In this model, the highest 25(OH)D concentration (77.3 nmol/L) was seen among ethnic Swedish women who had taken a sun holiday and the lowest among immigrants who had taken no sun holiday (25.2 nmol/L).

**Figure 2:**
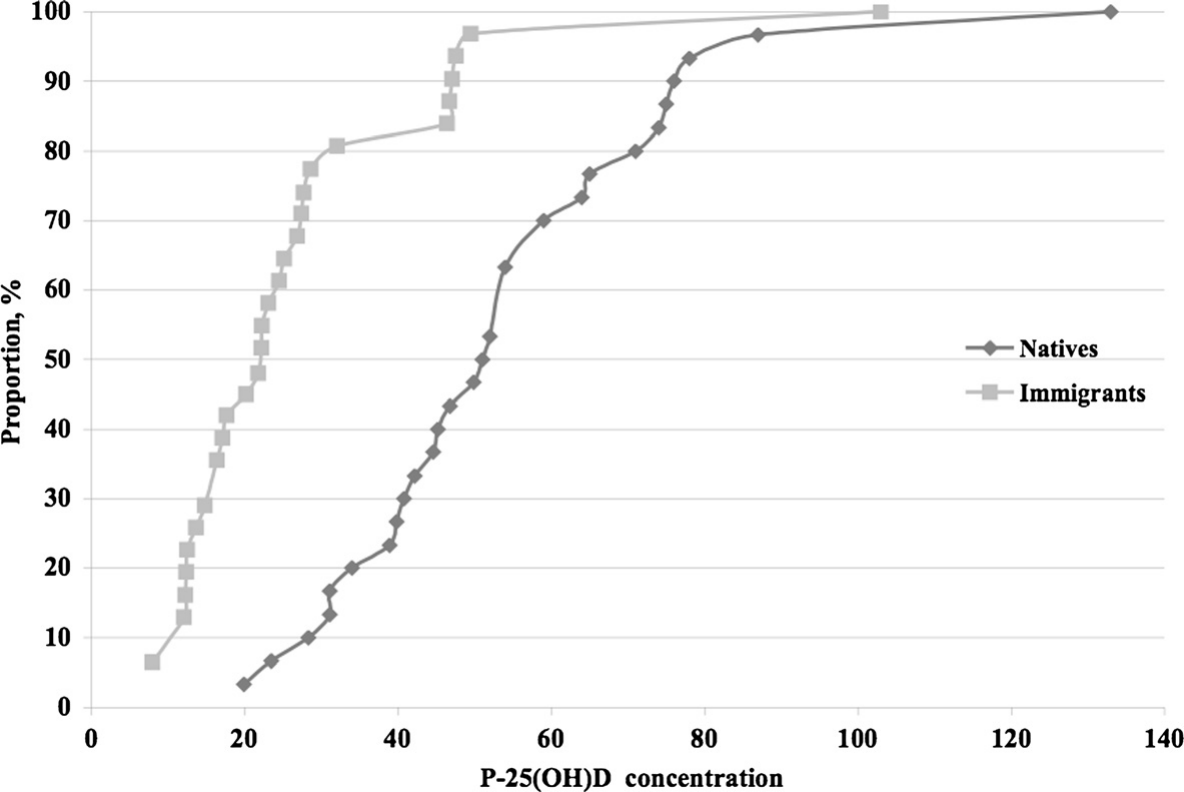
Cumulative distribution of plasma 25(OH)-vitamin D concentrations (P-25(OH)D) in native Swedish women and immigrant women.

The cumulative distribution of 25(OH)D concentrations by reported origin is shown in Figure 2, to show the shifts in distributions between groups and to display effects of other cut-off levels. 25(OH)D concentrations above 75 nmol/L were rare in the ethnic Swedish group. Fifty percent of the ethnic Swedish women had concentrations below 50 nmol/L. Sixty-one percent of the immigrants and 7% of the ethnic Swedish women had concentrations below 25 nmol/L. In the immigrant group only one woman had a concentration above 75 nmol/L, and she was later found to have received an injection of vitamin D in Iran 3 months earlier owing to pain problems.

**Figure 3:**
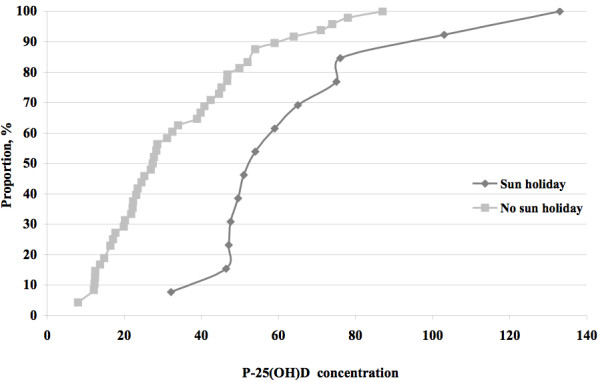
Cumulative distribution of plasma 25(OH)-vitamin D concentrations (P-25(OH)D) in women according to reported presence or absence of sun-holidays or not.

Similarly, Figure 3 shows the cumulative distribution of 25(OH)D concentrations in the two groups with sun holiday or no sun holiday: Among women who reported a sun holiday all had a 25(OH)D concentration >25 nmol/L. Among those who reported no sun holiday 44% had a concentration <25 nmol/L.

## Discussion

Overall, low concentrations of 25(OH)D were frequent, especially in immigrants. Reported sun holiday and ethnic Swedish status were the factors that positively, significantly and independently affected 25(OH)D concentrations in plasma, with sun holiday ranking highest. The non-significant effect of vitamin D intake from food and supplements as well as wearing of veil, was unexpected. Regarding vitamin D intake, it may reflect the overall low dietary intake of vitamin D.

Plasma concentrations of 25(OH)D are believed to reflect the vitamin D status in terms of production, absorption, and storage, with a half-life of 1-2 months [4]. In spite of this assumption, our results indicate a prolonged effect of a sun holiday. Whether this is due to the body being able to access stored vitamin D or whether other mechanisms provide an explanation is not known.

From a global point of view, vitamin D from sun exposure is said to be of most importance, but at latitudes around 60°N, where vitamin D is produced in the skin from sun irradiation only from April to September, it is not clear how large this contribution is [8,9]. In the present study, vitamin D intake from food and supplements was not always sufficient to avoid low plasma concentrations of 25(OH)D. In order to raise vitamin D in plasma addition of vitamin D through food, supplements or via increased production in the skin would be particularly important for women living at high latitudes.

Previous studies report that 25(OH)D concentrations <50 nmol/L are common in women living in the Norwegian arctic region (65-70ºN) during January and February [8]. The results of the present study with measurements between January and March are consistent with these observations, although our study is performed at more southern latitude (60ºN). Our data also concurs with results from epidemiological studies on immigrant populations in Norway and Denmark [9-13]. The importance of a sun holiday found in the present study accords with a study on elderly Swedish women from the general population [11], although our study population included younger patients, and from a primary health care setting [12,13].

Regular sun holidays seemed in this study to contribute more to high 25(OH)D concentrations than vitamin D through food and supplements. The findings suggest that sun holidays and immigrant status explain more variability in vitamin D levels than does food. Immigrant status might be anticipated to be confounded with socioeconomic factors such as income level, smoking habits and educational level. However, there was no difference in the number of sun holidays between immigrants and Swedes. As to vitamin D intake through food, since foods containing high amounts of vitamin D, such as egg yolk, fortified milk and margarines are relatively cheap, the choice of food is more likely to be dependent on culture, traditions and knowledge than to differences of income. There was no difference in the frequency of tobacco smoking between the two groups. Recommendations for sunbathing are also controversial, owing to the risk of skin cancer. However, the latter, may be less relevant for people with darker skin.

Frequently cited studies state that 15 minutes of sun irradiation per day is enough to obtain sufficient vitamin D [3]. However, these studies are based on individuals living further south and these results cannot be translated to apply to the situation at more northerly latitudes around 60°, where no vitamin D is produced in the skin from October to March, irrespective of hours of sunshine [14].

According to Webb and Engelsen [14], a rough calculation of the vitamin D production following sun exposure for three hours a day for seven days at latitude 40° leads to a total production of 4,500 μg vitamin D. Considering the fact that the half-life of vitamin D in the body is 2 months, and that the average time since the women had been on a sun holiday was six months, this would correspond to 500 μg being left at the time of the study. This circumstance would imply a substantial addition of vitamin D in the body.

Increasing interest in and knowledge about vitamin D deficiency in relation to multiple conditions [1,15,16], has led many physicians to test their patients’ vitamin D status. The high frequency of low concentrations of 25(OH)D in immigrant women in our study indicates that vitamin D deficiency may be common in female patients of immigrant origin in primary care. Vitamin D deficiency and insufficiency have by some researchers previously been considered to be uncommon in the female Swedish population [10], but these views are based on studies of postmenopausal women with osteoporosis. This observation might not be relevant for all women, and especially not for immigrant subpopulations.

For good bone health, blood concentrations of 50 nmol/L 25(OH)D are needed [1]. For other aspects of general health, a concentration of 75 nmol/L has been suggested [17]. Fifty percent of the ethnic Swedish women in our study had plasma 25(OH)D concentrations below 50 nmol/L. For immigrant women, the majority should probably be advised to take greater supplements of vitamin D than the present recommendations, and perhaps even without testing for vitamin D concentrations in the blood, especially if their intake from food is low. The latter could easily be assessed with a few questions since most vitamin D consumed in food is present in only a few food items.

The strengths of the study was that women with limited knowledge of the Swedish language were not excluded and that the individual interviews enabled inclusion of illiterate women, since they are a vulnerable population group with an increased risk of health problems [18]. Moreover, the study appointments took place between January and March in order to obtain blood samples that reflected the lowest values during the year in Sweden [19].

A limitation was that the study was too small to reliably rule out non-significant results and limited the generalizability. Also, the concept of sun holiday was difficult to standardize. In addition, the FFQ questionnaire has not been validated, but the results were compared with the results from a nationwide Swedish survey [20] and the intake of vitamin D was estimated to be similar (5.1 μg/d respectively 4.9 μg/d). Another limitation of this study may have been the choice of method for analysing plasma-25(OH)D. Other assays are available, and their comparability is uncertain. The HPLC method seems to be more accurate and reliable than other methods. In our study we found that two thirds of the immigrants had deficient concentrations of plasma-25(OH)D (<25 nmol/L), but if we had used another method, e.g. HPLC, probably fewer women would have had deficient concentrations. However, the difference in plasma-25(OH)D between our two groups would still remain.

In future research, the need of treatment for low 25(OH)D values in immigrants who have moved from southern to northern latitudes should be further investigated. Culturally adjusted measures should be considered for this vulnerable group.

## Conclusions

Vitamin D deficiency was common among the women in the present study, with sun holiday, origin as main determinants of 25(OH)D concentrations in plasma. Given a negative effect on health this would imply need for vitamin D treatment particularly in women who have moved from southern to northern latitudes.

## Abbreviations

25(OH)D: 25-OH-vitamin D; FFQ: Food frequency questionnaire.

## Competing interests

The authors declare that they have no competing interests.

## Authors’ contributions

AB, ÅA and GJ initially planned the study. AB and ÅA carried out the interviews and data collection. Statistical analyses were performed by GJ, AB and PK. All authors made substantial contribution to drafting the manuscript and revising it critically for important intellectual content. All authors have read and approved the final manuscript for submission.

## Authors’ information

Anne Björk, MD, works as a GP and is a part-time PhD student at the Family Medicine section at the Department of Public Health and Caring Sciences at Uppsala University, Sweden.

Åsa Andersson is a dietician at a primary health care centre in Uppsala, Sweden.

Karin Björkegren, MD, PhD, works as a GP and is also Senior lecturer at Uppsala University.

Annika Bardel, MD, PhD, works as a GP and is also lecturer at Uppsala University.

Gunnar Johansson, MD, PhD, is Professor of Family Medicine at Uppsala University.

Per Kristiansson, MD, PhD, works as a GP and is also Associate Professor of Family Medicine and lecturer at Uppsala University.

## Pre-publication history

The pre-publication history for this paper can be accessed here:

http://www.biomedcentral.com/1471-2296/14/129/prepub

## References

[B1] Ross AC Manson JE Abrams SA Aloia JF Brannon PM Clinton SK Durazo-Arvizu RA Gallagher JC Gallo RL Jones G The 2011 report on dietary reference intakes for calcium and vitamin D from the Institute of Medicine: what clinicians need to know J Clin Endocrinol Metabol 2011 96 1 53 58 10.1210/jc.2010-2704 PMC3046611 21118827

[B2] Lamberg-Allardt C Vitamin D in foods and as supplements Prog Biophys Mol Biol 2006 92 1 33 38 10.1016/j.pbiomolbio.2006.02.017 16618499

[B3] Holick MF Sunlight and vitamin D for bone health and prevention of autoimmune diseases, cancers, and cardiovascular disease Am J Clin Nutr 2004 80 6 Suppl 1678S 1688S 15585788 10.1093/ajcn/80.6.1678S

[B4] Holick MF Vitamin D status: measurement, interpretation, and clinical application Ann Epidemiol 2009 19 2 73 78 10.1016/j.annepidem.2007.12.001 18329892 PMC2665033

[B5] Områdesfakta http://www.uppsala.se/upload/Dokumentarkiv/Externt/Dokument/Om_kommunen/Omradesfakta/Gottsunda.pdf23998043

[B6] Fitzpatrick TB The validity and practicality of sun-reactive skin types I through VI Arch Dermatol 1988 124 6 869 871 10.1001/archderm.1988.01670060015008 3377516

[B7] Snellman G Melhus H Gedeborg R Byberg L Berglund L Wernroth L Michaelsson K Determining vitamin D status: a comparison between commercially available assays PLoS One 2010 5 7 e11555 10.1371/journal.pone.0011555 20644628 PMC2903481

[B8] Brustad M Alsaker E Engelsen O Aksnes L Lund E Vitamin D status of middle-aged women at 65-71 degrees N in relation to dietary intake and exposure to ultraviolet radiation Publ Health Nutr 2004 7 2 327 335 10.1079/PHN2003536 15003141

[B9] van Schoor NM Lips P Worldwide vitamin D status Best Pract Res Clin Endocrinol Metab 2011 25 4 671 680 10.1016/j.beem.2011.06.007 21872807

[B10] Lips P Duong T Oleksik A Black D Cummings S Cox D Nickelsen T A global study of vitamin D status and parathyroid function in postmenopausal women with osteoporosis: baseline data from the multiple outcomes of raloxifene evaluation clinical trial J Clin Endocrinol Metabol 2001 86 3 1212 1221 10.1210/jc.86.3.1212 11238511

[B11] Burgaz A Akesson A Oster A Michaelsson K Wolk A Associations of diet, supplement use, and ultraviolet B radiation exposure with vitamin D status in Swedish women during winter Am J Clin Nutr 2007 86 5 1399 1404 17991652 10.1093/ajcn/86.5.1399

[B12] Andersen R Molgaard C Skovgaard LT Brot C Cashman KD Jakobsen J Lamberg-Allardt C Ovesen L Pakistani immigrant children and adults in Denmark have severely low vitamin D status Eur J Clin Nutr 2008 62 5 625 634 10.1038/sj.ejcn.1602753 17440527

[B13] Holvik K Meyer HE Haug E Brunvand L Prevalence and predictors of vitamin D deficiency in five immigrant groups living in Oslo, Norway: the Oslo Immigrant Health Study Eur J Clin Nutr 2005 59 1 57 63 10.1038/sj.ejcn.1602033 15280907

[B14] Webb AR Engelsen O Calculated ultraviolet exposure levels for a healthy vitamin D status Photochem Photobiol 2006 82 6 1697 1703 16958558 10.1562/2005-09-01-RA-670

[B15] Holick MF Vitamin D deficiency New England J Med 2007 357 3 266 281 10.1056/NEJMra070553 17634462

[B16] Zittermann A Schleithoff SS Koerfer R Putting cardiovascular disease and vitamin D insufficiency into perspective Br J Nutr 2005 94 4 483 492 10.1079/BJN20051544 16197570

[B17] Holick MF Binkley NC Bischoff-Ferrari HA Gordon CM Hanley DA Heaney RP Murad MH Weaver CM Guidelines for preventing and treating vitamin D deficiency and insufficiency revisited J Clin Endocrinol Metabol 2012 97 4 1153 1158 10.1210/jc.2011-2601 22442274

[B18] Daryani A Berglund L Andersson A Kocturk T Becker W Vessby B Risk factors for coronary heart disease among immigrant women from Iran and Turkey, compared to women of Swedish ethnicity Ethn Dis 2005 15 2 213 220 15825967

[B19] Engelsen O The relationship between ultraviolet radiation exposure and vitamin D status Nutrients 2010 2 5 482 495 10.3390/nu2050482 22254036 PMC3257661

[B20] Becker W Pearson M Riksmaten 1997-1998 2002 Livsmedelsverket: Kostvanor och näringsintag i Sverige Uppsala

